# Scientific Evidence for Clinical Text Summarization Using Large Language Models: Scoping Review

**DOI:** 10.2196/68998

**Published:** 2025-05-15

**Authors:** Lydie Bednarczyk, Daniel Reichenpfader, Christophe Gaudet-Blavignac, Amon Kenna Ette, Jamil Zaghir, Yuanyuan Zheng, Adel Bensahla, Mina Bjelogrlic, Christian Lovis

**Affiliations:** 1 Division of Medical Information Sciences University Hospital of Geneva Geneva Switzerland; 2 Institute for Patient-centered Digital Health Bern University of Applied Sciences Biel Switzerland; 3 Faculty of Medicine University of Geneva Geneva Switzerland

**Keywords:** summarization, large language models, natural language processing, health care, electronic health records, scoping review, translational research, artificial intelligence

## Abstract

**Background:**

Information overload in electronic health records requires effective solutions to alleviate clinicians’ administrative tasks. Automatically summarizing clinical text has gained significant attention with the rise of large language models. While individual studies show optimism, a structured overview of the research landscape is lacking.

**Objective:**

This study aims to present the current state of the art on clinical text summarization using large language models, evaluate the level of evidence in existing research and assess the applicability of performance findings in clinical settings.

**Methods:**

This scoping review complied with the PRISMA-ScR (Preferred Reporting Items for Systematic Reviews and Meta-Analyses extension for Scoping Reviews) guidelines. Literature published between January 1, 2019, and June 18, 2024, was identified from 5 databases: PubMed, Embase, Web of Science, IEEE Xplore, and ACM Digital Library. Studies were excluded if they did not describe transformer-based models, did not focus on clinical text summarization, did not engage with free-text data, were not original research, were nonretrievable, were not peer-reviewed, or were not in English, French, Spanish, or German. Data related to study context and characteristics, scope of research, and evaluation methodologies were systematically collected and analyzed by 3 authors independently.

**Results:**

A total of 30 original studies were included in the analysis. All used observational retrospective designs, mainly using real patient data (n=28, 93%). The research landscape demonstrated a narrow research focus, often centered on summarizing radiology reports (n=17, 57%), primarily involving data from the intensive care unit (n=15, 50%) of US-based institutions (n=19, 73%), in English (n=26, 87%). This focus aligned with the frequent reliance on the open-source Medical Information Mart for Intensive Care dataset (n=15, 50%). Summarization methodologies predominantly involved abstractive approaches (n=17, 57%) on single-document inputs (n=4, 13%) with unstructured data (n=13, 43%), yet reporting on methodological details remained inconsistent across studies. Model selection involved both open-source models (n=26, 87%) and proprietary models (n=7, 23%). Evaluation frameworks were highly heterogeneous. All studies conducted internal validation, but external validation (n=2, 7%), failure analysis (n=6, 20%), and patient safety risks analysis (n=1, 3%) were infrequent, and none reported bias assessment. Most studies used both automated metrics and human evaluation (n=16, 53%), while 10 (33%) used only automated metrics, and 4 (13%) only human evaluation.

**Conclusions:**

Key barriers hinder the translation of current research into trustworthy, clinically valid applications. Current research remains exploratory and limited in scope, with many applications yet to be explored. Performance assessments often lack reliability, and clinical impact evaluations are insufficient raising concerns about model utility, safety, fairness, and data privacy. Advancing the field requires more robust evaluation frameworks, a broader research scope, and a stronger focus on real-world applicability.

## Introduction

In February 2024, Van Veen et al [1] reported that large language models (LLMs) could outperform medical experts in clinical text summarization. Their work investigated the effectiveness of specifically tailored models to accurately summarize clinical documents. However, a careful analysis of the experimental design and the evaluation methodology questions this statement.

Clinical text summarization is described by Keszthelyi et al [2] as the art of collecting, synthesizing, and communicating patient information. An effective summary must be tailored to meet the needs of its intended audience, which requires a clear definition of the clinical text summary’s purpose to ensure relevance and meaning.

In the fast-paced environment of modern health care, coupled with information overload in electronic health records (EHRs), physicians face added cognitive load and time pressure. Misunderstandings, incomplete information sharing, or delays in conveying critical patient details ultimately affect the quality of care and decision-making [3]. Thus, reducing the administrative burden on clinicians has become a critical need.

**Figure 1:**
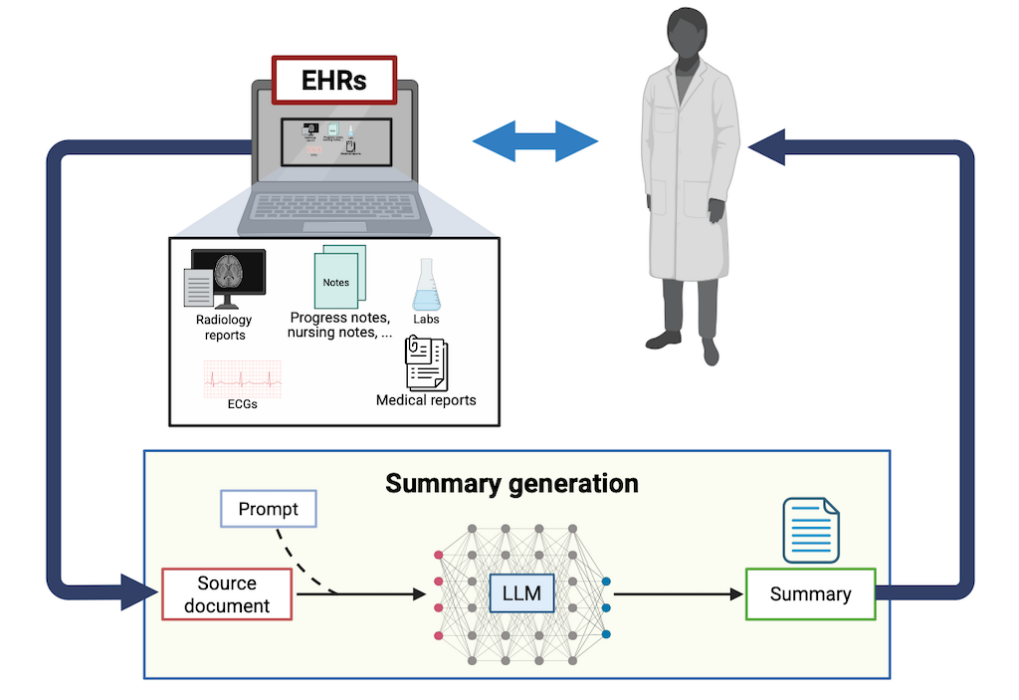
The process of generating clinical summaries using LLMs. Source documents from EHRs, such as radiology reports, progress notes, nursing notes, laboratory test results, and medical reports, are processed by an LLM to generate a summary intended, in this case, for a physician. EHR: electronic health record; LLM: large language model. Created in BioRender (Bednarczyk, L., 2025, https://BioRender.com/trqp263; [12]).

LLMs can process significant volumes of input data and produce coherent output text [4,5]. As such, they present an opportunity to alleviate clinicians’ administrative workload by summarizing patient information contained in EHRs. While ensuring strict adherence to data privacy standards, effective models could deliver context-specific summaries that meet clinical objectives. Potential applications include optimizing information retrieval, as critical data are often buried within extensive, noisy, and repetitive entries [6,7]; or automating summarization tasks that are traditionally carried out manually, such as discharge summaries [8-10] (Figure 1). Additionally, patient-directed simplified reports could support informed decision-making [10,11].

Several researchers have examined the application of LLMs in health care [13-17]. Bedi et al [15], Wang et al [17], Park et al [14], and Pressman et al [16] all reported concerns regarding the strategies used to evaluate these models. Meng et al [13] highlighted the lack of robust clinical studies to validate LLMs’ effectiveness and safety in real-world settings.

To the best of our knowledge, no comprehensive review has specifically addressed the performance of LLMs in clinical text summarization. This gap raises critical questions: Do LLMs genuinely outperform medical experts in summarizing clinical texts? Can they be used with confidence in clinical settings for summarization tasks?

This review seeks to assess the reliability of performance findings and their applicability to health care settings through a detailed analysis of the literature, including insights from studies such as those discussed by Van Veen et al [1].

The specific research objectives are as follows.

- Present the current state of research on clinical text summarization using LLMs.
- Evaluate the level of evidence in the current state of research.
- Assess whether these models can be used with confidence in clinical settings.
- Provide expert recommendations for current and future research.

## Methods

### Study Design

This scoping review focused on the summarization of clinical text within EHRs using LLMs. The authors adhered to the PRISMA-ScR (Preferred Reporting Items for Systematic Reviews and Meta-Analyses extension for Scoping Reviews) checklist (Multimedia Appendix 1).

### Search Strategy

Relevant literature, published between January 1, 2019, and June 18, 2024, was identified from 5 databases: PubMed, Embase, Web of Science, IEEE Xplore, and ACM Digital Library. The search strategy was structured around three key dimensions: “summarization,” “large language models,” and “healthcare,” which were derived from the above-mentioned research objectives and combined with Boolean operators. Related search terms and exact database queries are presented in Multimedia Appendix 2.

### Eligibility Criteria

Eligibility criteria (Textbox 1), defined prior to the screening process and agreed upon by 2 authors (DR and LB), were framed as exclusion criteria to ensure a comprehensive identification of all relevant papers. Studies that did not describe a model based on the original transformer architecture as introduced by Vaswani et al [18], did not focus on clinical text summarization, or did not engage with free-text data were excluded. We also excluded publications that were not original research, including editorials, reviews, or comments, as well as those that were not retrievable. Only peer-reviewed literature in English, French, Spanish, or German was considered. January 1, 2019, was chosen as the cutoff date based on existing literature [19].

**Eligibility criteria.**

Exclusion criteria

- The source of evidence (SOE) does not describe a model based on the original transformer architecture.
- The SOE does not describe clinical text summarization or summarize medical texts that are not clinical (eg, biomedical texts or medical evidence summarization).
- The SOE does not deal with free-text data.
- The SOE is published before January 1, 2019.
- The SOE does not describe original research.
- The SOE is not published in English, French, Spanish, or German.
- The SOE is an editorial, review, or comment.
- The SOE is not retrievable.
- The SOE is not peer-reviewed.

### Screening Process

During the initial quality check, a random sample of 10 studies, including titles and abstracts, were screened by 2 authors (LB and DR) to refine exclusion criteria and ensure consistency in screening. Discrepancies were resolved collaboratively.

The screening process was then conducted in 2 stages by the same 2 authors (LB and DR). In the first stage, titles and abstracts of the remaining studies were independently screened, with any conflicting decisions defaulting to eligibility to prevent premature exclusion of potentially relevant studies. In the second stage, full-text screening was performed independently by both authors, with any disagreements resolved through discussion and consensus.

### Data Synthesis

Before data extraction, a second quality improvement phase was conducted. Two authors (LB and DR) independently extracted data from a random sample of 3 studies to refine and finalize the data extraction table. Subsequently, full-text screening and data extraction were independently carried out by three authors (LB, CGB, and AKE) using a predefined spreadsheet.

Data extraction was organized into three main aspects: (1) study context and characteristics, (2) scope of research, and (3) evaluation methodologies.

- Study context and characteristics included the year and location of publication (based on the corresponding author’s address), the type of journal, the study design (inferred based on the described methodology, distinguishing between retrospective and prospective approaches), and the type of dataset used categorized as real patient data (open-source or proprietary) or synthetic data.
- The scope of research encompassed information related to the field of application, the summary intention, summarizing techniques used, technological aspects, and ethical considerations. The field of application refers to the domain where the summarization methods were developed and evaluated, covering department, country, patient demographics, and language coverage. Department and country details were extracted directly from the dataset information. Summary intention refers to the purpose of the summary, defined in this work based on the target audience, the summarization objective, and the source document. The summarization technique covered the details of input documents and the summarization techniques used. Technological aspects included relevant modeling characteristics (pretraining, fine-tuning strategies, and prompt engineering) approaches, deployment environments (eg, on-premises, cloud-based), hardware requirements, and associated computational costs. Ethical considerations included dataset deidentification and the reporting of institutional review board (IRB) approval.
- Evaluation methodologies included the strategies used, sample sizes, metrics, and additional details on each evaluation framework used.

Any discrepancies in data extraction were resolved through discussion with a fourth author (DR) to ensure accuracy and consistency throughout the process. The completed data extraction is provided in Multimedia Appendix 3 [1,21-49].

No assumption was made about missing or unclear details unless explicitly stated. The authors of the included studies were not contacted for clarification. The extraction focused exclusively on information pertinent to the summarization of clinical text found in the EHR.

Extracted data were synthesized using a descriptive approach, complemented by narrative synthesis, and presented in tables and figures where applicable. Data were summarized and described according to the 3 categories of data extraction as described earlier. The synthesis aimed to clearly outline key trends and characteristics across the included studies without conducting statistical analyses or quantitative meta-analyses.

## Results

### Study Contexts and Characteristics

**Figure 2:**
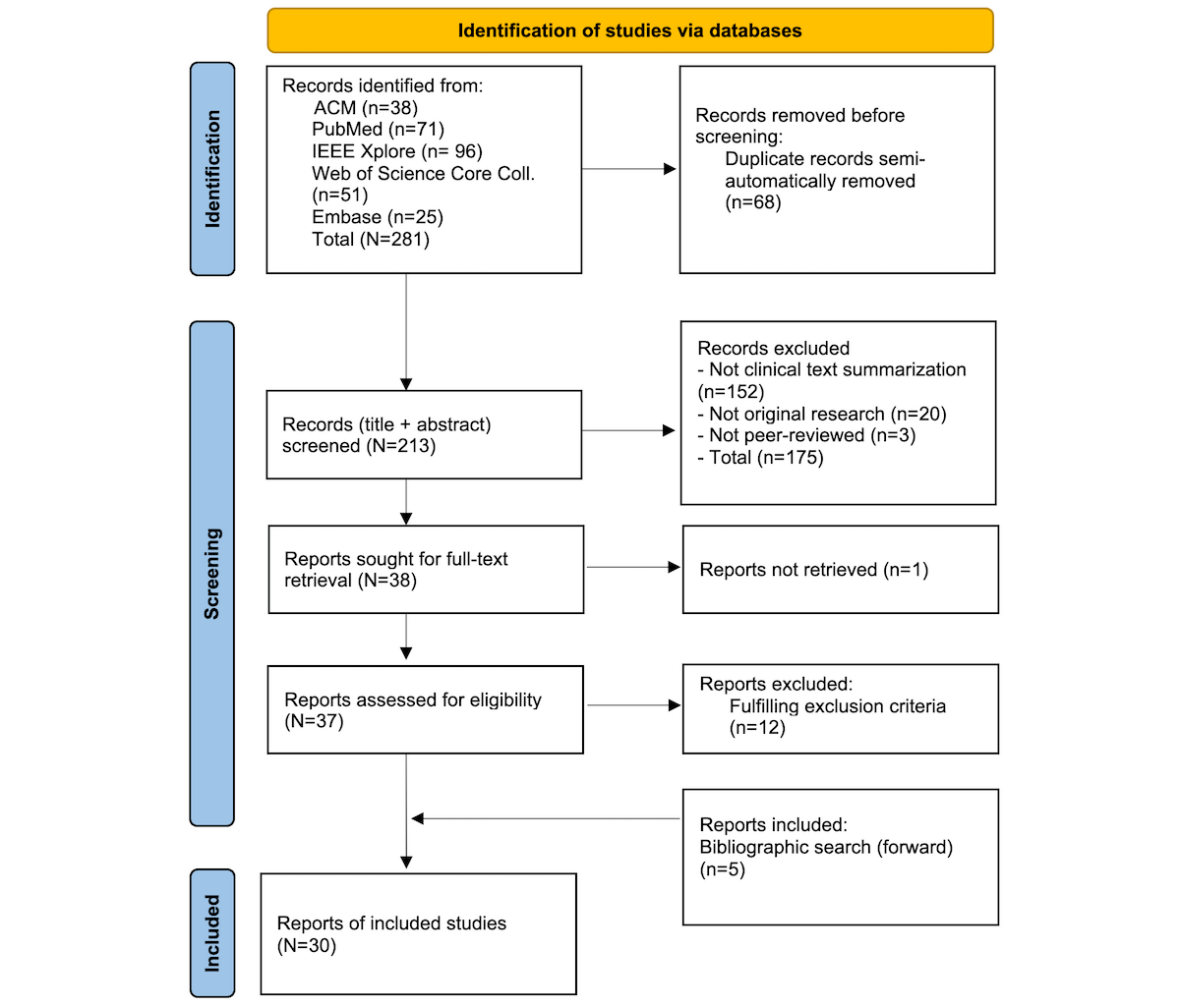
PRISMA (Preferred Reporting Items for Systematic Reviews and Meta-Analyses) flow chart.

This scoping review included a total of 30 studies. Of the 281 retrieved by database queries, 25 were deemed eligible following title, abstract, and full-text screening. Additionally, 5 studies were incorporated through manual reference screening using the snowballing technique [20]. An overview of the literature retrieval and screening process is presented in the PRISMA (Preferred Reporting Items for Systematic Reviews and Meta-Analyses) flowchart (Figure 2).

**Figure 3:**
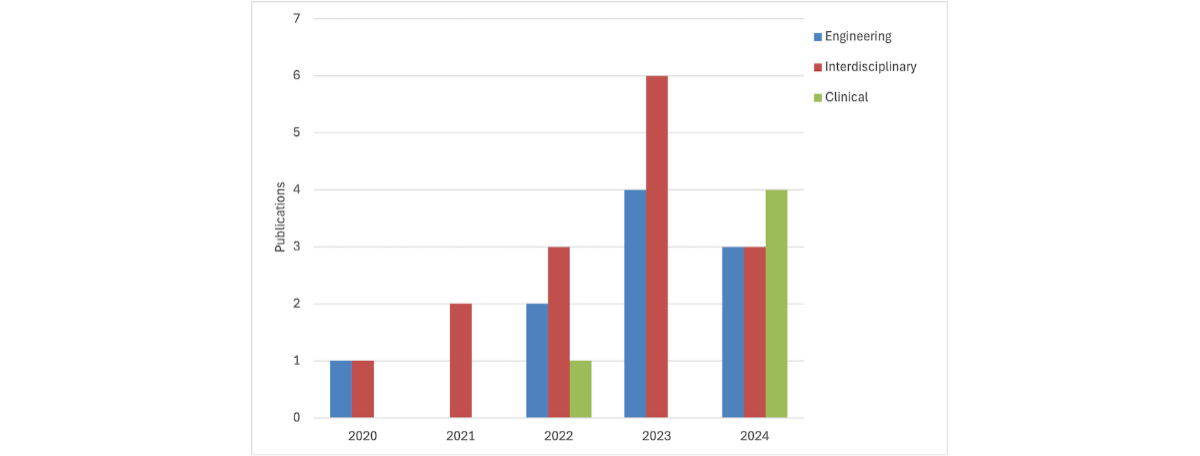
Annual publication count categorized by journal type.

Publications were distributed across interdisciplinary (n=15, 50%), engineering (n=10, 33%), and clinical (n=5, 17%) journals. The annual publication count showed an exponential growth trend, with no study published prior to 2020 (Figure 3). Clinical document summarization was the primary research objective in the majority of studies (n=27, 97%), while Li et al [21] used summarization as a preprocessing step in sepsis prediction.

The study location was dominated by the United States (n=10, 33%), followed by China (n=5, 17%), India (n=3, 10%) [22-24], and the United Kingdom (n=2, 7%) [25,26]. Other locations included Australia [27], France [28], Germany [29], Lebanon [30], Spain [31], and Taiwan [32] (n=1, 3% each), and Jiang et al [33] reported multiple corresponding authors from different countries. For 10% of studies (n=3), the corresponding author’s address could not be determined [34-36].

Among the studies, all used an observational retrospective design, using existing patient data to evaluate model performance. 93% used real patient data (n=28), primarily from open-source datasets (n=18, 60%), with the Medical Information Mart for Intensive Care (MIMIC) series (n=15, 50%) and the Indiana University X-Ray database (n=8, 27%) being the most reported (Table 1). Additionally, 47% of studies used proprietary databases (n=14). Goswami et al [22] mentioned using real patient data but did not specify the dataset used. Caterson et al [25] and Wu et al [28] used synthetic data, relying on scenarios generated by the authors.

**Table 1:** Distribution of specific open-source datasets.

Open-source dataset	Publications, n (%)	
MIMIC^a^	15 (50)	
IU X-Ray^b^	8 (27)	
KCH^c^	1 (3)	
SAM^d^	1 (3)	
Stanford Coll^e^	1 (3)	
PubMed	1 (3)	

^a^MIMIC: Medical Information Mart for Intensive Care.

^b^IU-XRAY: Indiana University X-Ray database.

^c^KCH: King’s College Hospital database.

^d^SAM: SAMSum corpus.

^e^Stanford Coll: Stanford Hospital Collection.

### Scope of Research

#### Field of Application

Clinical departments in which the models were evaluated could be retrieved in 22 studies (73%) and included mainly the intensive care unit (ICU; n=15, 50%) neurology (n=2, 7%) [26,37], and oncology (n=2, 7%) [28,29]. Other departments included cardiology [38], geriatrics [27], neurosurgery [39], and orthopedics [25] (n=1, 3% each). Chen et al [32] and Vinod et al [24] both addressed multiple areas.

Dataset geographical origin was identified in 28 studies (93%). It was predominantly the United States (n=19, 63%) and China (n=3/30, 10%) [33,41,43]. Other origins included Germany [29], the United Kingdom [26], Taiwan [32], Spain [31], and Australia [27] (n=1, 3% each). Additionally, Searle et al [26] included patients from 2 different countries. Four studies (13%) reported on patient demographics: 3 (10%) provided information on sex, race, and ethnicity [21,39,42], and López et al [31] on the sex-age ratio of the study population.

The language coverage was mainly English (n=26, 87%), including 3 (n=3, 10%) studies explicitly stating it and 23 (n=23, 77%) inferred based on dataset sources. Additionally, 3 studies (10%) addressed the summarization of clinical documents in Chinese [33,41,43], and 1 (3%) in German [29].

#### Summary Intention

The intended audience of the generated summary was specified in 16 (53%) studies. Most studies targeted health care professionals (n=12, 40%), while a smaller proportion focused on patients (n=2, 7%) [30,44]. In addition, 2 studies (n=2, 7%) aimed to serve both patients and health care professionals [22,25]. Li et al [21] used summarization as a preprocessing step for subsequent modeling tasks to enhance sepsis prediction, rendering the identification of a target audience irrelevant.

Most studies (n=27, 90%) focused on a single summarization task while only a few, such as Alkhalaf et al [27], Van Veen et al [1], and Zhu et al [40], explored multiple summarization tasks. The details of summarization objectives and input sources used in each study are provided in Multimedia Appendix 4 [1,21-49].

Summarization objectives included mainly generating the impression section of radiology reports (n=12, 40%), followed by generating the hospital course section of discharge summaries (n=3, 10%). Notably, the description of the summarization objectives varied across studies. Some specified the exact section of a document to be generated (eg, the impression section of radiology reports), while others described the types of source documents and the key information to be extracted [24].

Regarding input sources, most studies (n=19, 73%) used a single type of text corpus, with radiology reports being the most common (n=17, 57%), followed by progress notes (n=2, 7%) and patient forms (n=1, 3%). Overall, 23% (n=7) of studies used multiple-type text corpora. Furthermore, 13% (n=4) did not explicitly specify their source [22,26,32,44].

#### Summarization Methodology

**Figure 4:**
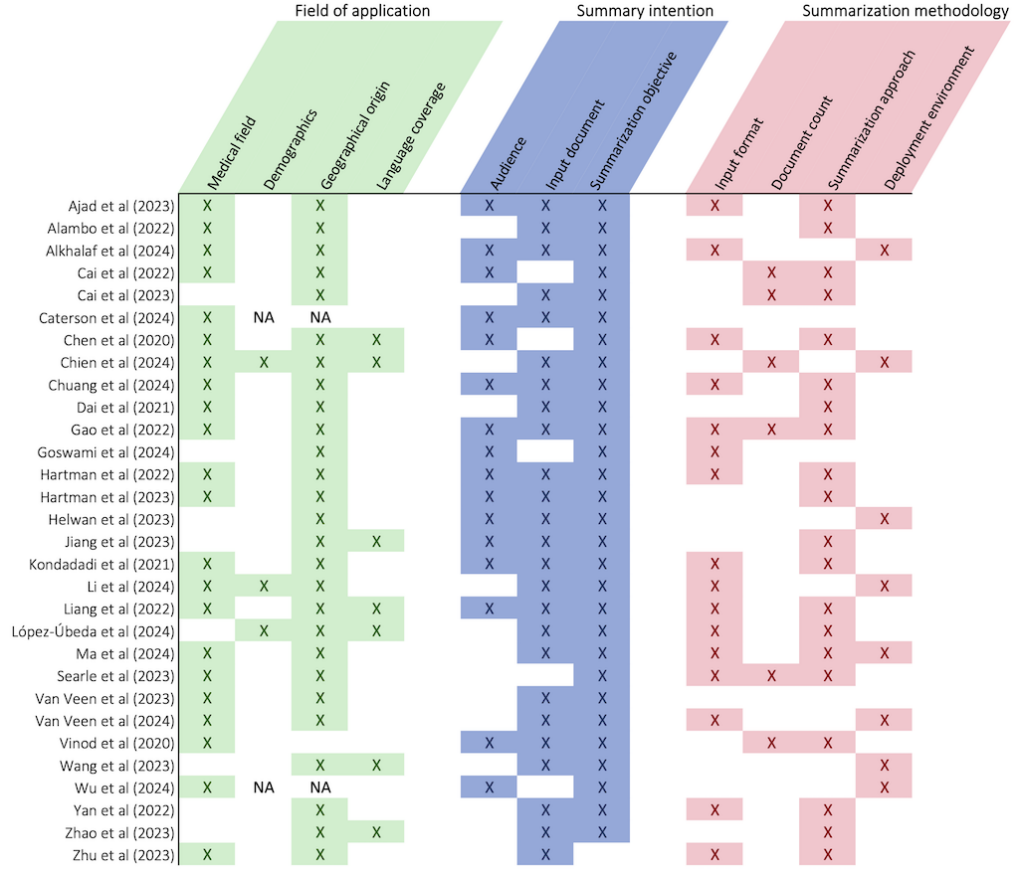
Overview of the reported information, including the medical field, dataset demographics, geographical origin of the test set, language coverage of the study, the intended audience of the summary (eg, physician, patient), a document used as input, summarization objective, input format (structured, unstructured, and both), input document count (single, multiple, and single-multiple), summarization approach (abstractive, extractive, and hybrid), and deployment environment (on-premises and cloud-based) [1,21-49]. NA: not available.

Fifteen (50%) studies explicitly mentioned the input source structure: 43% (n=13) used unstructured data only, while 7% (n=2) also reported using structured data such as patient demographics [27,45]. See Figure 4 for an overview of the reported information on experimental design. Six (20%) studies explicitly reported on the number of documents used as input at once: 4 (13%) mentioned single-document summarization, Searle et al [26] specified multidocument summarization, where the model took a cluster of related documents as input, and Chien et al [39] referred to a single-multiple document approach, where multiple documents were combined and treated as a single input. 20 publications (67%) specified the summarization technique used: 17 (57%) used abstractive methods, 7 (23%) extractive methods, and 2 (7%) hybrid methods [29,37]. Among these, 5 (17%) evaluated at least 2 approaches. While abstractive summarization generates new sentences that paraphrase the core ideas of the source text, extractive summarization selects and compiles existing key sentences or phrases directly from the original content [50].

Twenty-six (87%) studies reported on open-source models, with bidirectional encoder representations from transformers–based models being the most frequently cited (n=12, 40%). Seven (23%) studies investigated proprietary models, all based on commercial services provided by OpenAI. Two (7%) examined both model types [1,39], while Li et al [21] used an ensemble model. Seven (23%) studies performed additional pretraining of existing model architectures prior to fine-tuning. Furthermore, 23 (77%) mentioned fine-tuning. Ten (33%) studies conducted prompt engineering.

Eight (27%) studies reported on the deployment environment: 3 (10%) indicated on-premise deployment [27,30,39], and 6 (20%) the use of external services, including Azure OpenAI application programming interface (API) [1], and OpenAI API [21,28,36,39,41]. Sixteen (53%) studies reported on hardware requirements, whereas none mentioned associated costs.

### Ethical Considerations

Deidentification was conducted in 13 (43%) studies, and anonymization in 2 (7%) studies. Additionally, Wang et al [41] mentioned “data desensitization,” and López et al [31] mentioned ensuring that no patient or doctor information was included in datasets. IRB approval was reported in 30% (n=9) of studies.

### Evaluation Methodology

#### Evaluation Approaches

External validation, which involves testing models on datasets not used during training, was not explicitly reported in most cases. However, 2 (7%) studies reported testing on unseen datasets during model development (training or fine-tuning) and relied solely on automated metrics for external evaluation [35,46]. Internal validation, which assesses a model’s performance using a dedicated sample of the dataset it was trained on, was conducted in all studies.

Global performance evaluation, defined as the evaluation process conducted on the full test set, was conducted was conducted all studies. Furthermore, 16 (53%) studies used both automatic validation metrics and human evaluations, while 10 (33%) used automatic metrics only, and 4 (13%) human evaluations only. Subgroup performance evaluation was reported in 2 (7%) papers. Out-of-distribution performance testing, a process that evaluates how well a model performs on data that are underrepresented in the training set [51], was reported by Van Veen et al [47]. Subcategory testing was reported by Liang et al [29], who evaluated the impact of different stages of cancer on model performance using patient degree matching.

Model failure analysis was conducted through error categorization in 6 (20%) studies. Four (13%) studies addressed both the classification and quantification of error and 2 (7%) focused exclusively on error classification [44,47]. Bias analysis remained unexplored, as no studies explicitly report structured bias assessments. Finally, patient safety risk analysis was assessed by Van Veen et al [1] (n=1, 3%) using a Healthcare Adapted Risk Management Scale [1].

A broader understanding of model performance was achieved through various approaches, including ablation studies (n=8, 27%) and attention distribution analysis (n=2, 7%) [33,34]. Additionally, Zhao et al [43] included analyses of sparsity, loss curves, and the Zipf distribution, while Li et al [21] evaluated model performance through a proxy task involving sepsis prediction.

#### Automated Performance Metrics

Automated performance metrics were reported in 26 (87%) papers. Test set size, mentioned in 23 (77%) studies, included mainly between 15 and 1000 documents (0-1000 documents: 33%; 1000–5000 documents: 30%; more than 5000 documents: 10%). Randomized sampling was explicitly mentioned in 5 (17%) publications, while 3 used the entire dataset as a test set (10%) [25,39,42].

Reference-based metrics were widely used (n=25, 77%). Specifically, this type of metrics compared generated summaries to predefined reference summaries that encompassed the original document (n=18, 60%), manually labeled data (n=4, 13%), and expert-generated summaries (n=2, 7%) [1,39]. Two (7%) studies did not mention the reference summary used [22,26]. Validation of reference summaries was conducted in 7 (23%) studies, including 1 (3%) using the original document as a reference summary [43]. Recall-Oriented Understudy for Gisting Evaluation score was the most frequent performance measure (n=24, 80%), followed by Bilingual Evaluation Understudy (n=8, 27%) and bidirectional encoder representations from transformers score (n=7, 23%).

The test set count and evaluation metrics for both automatic metrics and human evaluation in each publication are detailed in Multimedia Appendix 5 [1,21-49].

#### Human Evaluation

Human assessment was reported in 20 (67%) papers. Test set size, mentioned in 18 (60%) studies, included mainly between 2 and 50 documents (2-50 documents: n=10, 33%; 50–100 documents: n=6, 20%; more than 100 documents: n=2, 7%). Randomized sampling was explicitly mentioned in 12 (40%) publications, including Li et al [21] who specified the use of 5-fold cross-validation. Additionally, 3 (10%) studies used the entire dataset [25,39,41].

Metrics, detailed in 18 (60%) studies, encompassed readability (n=12, 40%), factual correctness (n=12, 40%), and the adequacy of provided information directly related to the summary intention (n=12, 40%). These assessments include relevance [26,31], completeness [1,37] (n=2, 7% each), the ability to capture critical information [47], adequacy [44], informativeness [34], omission or insertion [25], comprehensiveness [39], and effectiveness [24] (n=1, 3% each). Additionally, clinical use was assessed in 3 (10%) studies and involved estimated clinical time saved [28], ease of revision [44], and clinical use [43].

Blind analysis was reported in 6 (20%) publications. The assessor recruitment process was documented by Jiang et al [33] and Li et al [21] (n=2, 7%), who specified including volunteers and invitees respectively. Assessors’ affiliation, reported in 16 (53%) studies varied. Most studies (n=14, 47%) included at least 1 in-domain physician. The inclusion of several participants in the manual review process was common (n=14, 47%), with 9 (30%) studies specifically involving at least 2 in-domain physicians.

Ten studies (33%, 10/30) reported at least 2 raters per document. Interannotator agreement score was reported in 4 (13%) studies and included intraclass correlation [1,37], Pearson *r* of human evaluation scores [44], and Cohen κ [26]. The use of measurement scales, documented in 14 papers (47%), involved mainly numeric scores (n=7, 23%) and Likert Scale (n=6, 20%). Additionally, Cai et al [44] used a scoring scale [44].

Three (10%) studies mentioned following a specific protocol for human evaluation: Chien et al [39] applied the approach proposed by Goldstein et al [52], and Lopez et al [31] and Searle et al [26] followed the protocol SummEval proposed by Fabbri et al [53]. The study setup was documented in 6 (20%) studies, where authors described the tools used in the research process, such as interfaces and questionnaires.

Summary ranking methods were primarily independent (n=19, 63%), where items are evaluated independently without direct comparison to one another. In contrast, Lopez et al [31] used a pairwise comparison approach, where items are evaluated by directly comparing 2 options at a time, allowing for a more relative assessment of rankings.

## Discussion

### Principal Findings

This scoping review offers a comprehensive overview of the state of research on clinical text summarization using LLMs. Concerns have been raised about the applicability of certain machine learning models in clinical settings, where their effectiveness failed to meet real-world expectations [54-56]. Our analysis suggests that similar challenges may extend to the field of automated summarization. The following sections discuss findings, highlight key barriers in translating research findings into practical clinical applications, and propose directions for future research.

#### A Narrow Research Scope

Current research focuses on a limited range of summarization objectives, patient populations, and medical specialties. Most studies (n=17, 57%) focused on radiology reports, particularly the impression section (n=15, 50%). Study populations were predominantly ICU patients (n=15, 50%), from US-based institutions (n=19, 73%). Language coverage was predominantly English (n=26, 87%). MIMIC, an open-source dataset originating from ICU from Beth Israel Deaconess Medical Center was used in 50% of studies (Figure 4) [57].

The limited research scope, combined with the heavy reliance on a few publicly available datasets, raises concerns about whether research is driven by clinical needs or data availability. ICU patients constitute only a small subset of the broader health care population encountered in routine clinical practice, limiting the generalizability of findings. The practical necessity of automating the impression section in radiology reports is debatable [58].

Additionally, open-source datasets fail to capture real-world complexities such as variations in writing styles, clinical workflows, and patient populations [59,60]. As a result, many potential applications remain yet unexplored. Future research should ensure alignment with clinical needs, and expand its scope to include a wider range of use cases, medical specialties, patient populations, and language coverage (Textbox 2).

**Key research priorities in study elaboration for large language model (LLM)–based summarization. This textbox highlights critical areas for refining and expanding research methodologies to improve clinical validity, ethical and legal compliance, and practical deployment of summarization models in health care.**

**Research objectives**

- Ensure research objectives directly address real-world clinical challenges.
- Expand the scope of investigation by diversifying use cases, medical specialties, and patient populations to enhance practical applicability.
- Clearly define the context of summarization objectives, including purpose, target audience, and expected outcomes, to ensure a clinically valid evaluation.

**Summarization methodology**

- Expand knowledge of both the performance and limitations of LLMs by evaluating their ability to handle real-world complexities, such as multisource summarization.

**Model performance and clinical impact evaluation**

- Conduct a context-aware validation of the model (or task-specific evaluation) through human assessment.
- Assess model generalizability via external validation, subcategory testing, and k-fold cross-validation.
- Evaluate model utility by analyzing its impact on the intended audience to determine whether summarization systems effectively fulfill their clinical purpose.
- Perform failure analysis and subsequent patient safety risk assessment to evaluate the clinical impact of identified errors on patient safety.
- Conduct bias analysis by assessing outputs for discriminative language, including bias related to gender, race, socioeconomic status, substance use, and mental health, to ensure fairness.

**Address data security and privacy**

- Ensure compliance with legal agreements, and implement data protection strategies such as on-premises deployment to safeguard patient privacy and regulatory standards.

Another limitation observed in this research is the lack of a clear description of summarization objectives. For example, Zhu et al [40] described objectives as “to generate a few significant impressions” or “to generate a few critical diagnosis results” (Multimedia Appendix 4 [1,21-49]). These descriptions lack precision, making it difficult to assess what constitutes a “significant” or “critical” diagnosis result, and how the output should be evaluated. As such, comparing studies and assessing model effectiveness becomes difficult.

Poorly defined research objectives can also lead to irrelevant research or fail to ensure proper model validation. For example, Helwan et al [30] identified patients as the target audience and summarizing and simplifying radiology reports as the summarization objective. In contrast, their application focused on generating the impression section of radiology reports primarily meant for communication between the ordering physician and the radiologist [61]. This misalignment between the stated objective and the actual application further highlights the need for greater clarity in research goals.

Future research should define summarization objectives, specifying purpose, target audience, and expected outcomes, to ensure a clinically relevant evaluation by aligning assessment criteria with intended clinical applications (Textbox 2). Additionally, studies should provide a detailed text corpus description, ensuring clear and consistent terminology across regions, disciplines, and institutions to facilitate comparability across studies (Textbox 3).

**Reporting recommendations specific to the large language model (LLM)–based summarization research.**

**Research objective**

- Provide a thorough description of the summarization objective studied, a precise understanding of its purpose, intended audience, and expected outcome.

**Summarization methodology and model evaluation**

- Provide a clear and detailed description of the text corpus used, ensuring that terminology is well-defined and understandable across different regions, disciplines, and institutions.
- Specify the structure of the input test data (structured and unstructured).
- Report the number of documents processed by the model (single, multiple, and multiple-single).
- Detail the summarization technique used (abstractive, extractive, and hybrid).
- Specify the model deployment environment (on-premises and externally).
- Provide demographic information on the dataset used for LLM development, tuning, or evaluation.

**Technological and cost requirements**

- Provide a detailed report on the computational resources needed for model deployment and outline the associated costs.

#### Limitations in Addressing Real-World Effectiveness

Most studies used abstractive summarization (n=17, 57%), typically with single-document inputs (n=4, 13%) and unstructured data (n=13, 43%). In terms of deployment methods, 10% (n=3) of models deployed on-premises and 20% (n=6) used external cloud services. Furthermore, as depicted in Figure 4, notable underreporting across studies was noted.

These findings highlight concerns about model effectiveness, particularly in synthesizing multisource data and handling longitudinal records. In clinical practice, physicians summarize patient data from single or multiple sources using different perspectives for decision-making, communication, or documentation. However, only Searle et al [26] specified multidocument summarization, while Chien et al [39] mentioned a single-multiple document approach. Multidocument summarization presents additional challenges, such as maintaining coherence, reducing redundancy, and ensuring consistency [62,63]. Furthermore, temporality remains a key obstacle, as clinical narratives span longitudinal records. Chien et al [39] suggested that overcoming this challenge may require dedicated models for temporal relation extraction to accurately capture event sequencing. Thus, the full spectrum of summarization performance remains largely unexplored. Future research should deepen insights into LLM strengths and weaknesses by assessing their ability to manage real-world challenges, including multisource summarization (Textbox 2).

Additionally, concerns persist regarding the real-world applicability of current summarization models due to deployment constraints. On-premise deployment presents hardware limitations that must be addressed to maintain optimal performance and accuracy, whereas cloud-based models necessitate stringent data privacy measures and compliance with regulatory frameworks [64,65]. Without systematic reporting on these deployment considerations, it remains uncertain whether summarization models can be practically integrated into health care workflows. Identifying practical strategies for real-world implementation is essential to bridge the gap between research and clinical application.

Finally, the widespread under-reporting across studies undermines the validity of performance assessments (Figure 4), making it difficult to compare models, reproduce results, and evaluate their real-world feasibility. To address this, future research must adopt comprehensive reporting guidelines, such as the Transparent Reporting of a multivariable prediction model for Individual Prognosis or Diagnosis (TRIPOD)+LLM checklist, which extends the original TRIPOD guidelines to ensure standardized reporting in LLM-based health care research [66]. Additionally, Textbox 3 outlines reporting recommendations specific to summarization research. Enhancing transparency in reporting will improve integrity, enable meaningful cross-study comparisons, and ultimately support the development of summarization models that are both clinically relevant and practically deployable.

#### Challenges and Limitations in Model Evaluation

Evaluation frameworks varied widely in used strategies and metrics (Multimedia Appendix 5 [1,21-49]). All studies conducted internal validation, primarily with automatic metrics (n=26, 87%), while 67% (n=20) included human evaluation. Few studies conducted external validation (n=2, 7%), failure analysis (n=6, 20%), or patient safety risk assessments (n=1, 3%), and none performed bias evaluation.

#### Challenges in Ensuring Reliable Evaluations

Effectively evaluating generative outputs in summarization tasks remains a challenge, as it requires evaluating both textual quality and contextual appropriateness [66]. Human evaluation remains the gold standard for text summarization, offering the contextual insight necessary for accurate summary assessment [67-69]. Automatic validation metrics, however, lack contextual understanding and therefore cannot reliably determine relevance, correlate poorly with human judgment, and are considered underinformative [67,70].

Findings suggest that a notable portion of studies (33%) may lack a clinically meaningful assessment of model performance, as they relied solely on automatic validation metrics. Several studies reported limitations of automatic validation metrics in summary evaluation. Cai et al [44] and Hartmann et al [45] noted that these metrics were not adequate to capture summary quality, while Liang et al [29] and Zhao et al [43] reported that these metrics did not evaluate the clinical validity (or usability) of summaries. As no consensus is reached on the reliability of automatic validation metrics in evaluating abstractive summarization, several studies have proposed combining automatic metrics with human evaluation and conducting correlation analyses to better assess their validity and practical value [66].

Further limitations were observed among the 20 studies conducting human evaluations: 15% (3/20) did not report the number of assessors [22,27,43], 20% (4/20) did not specify the assessor’s affiliation [22,27,41,43], 90% (18/20) did not specify their assessors’ recruitment processes, and 70% (14/20) of studies lacked blinded analysis. While 50% (10/20) of studies reported dual annotations, only 4 out of 20 (20%) reported interannotator agreement. Addressing these issues is essential to ensure reliable performance assessment. While yet no standardized best practices for human evaluation in text summarization currently exist, research in this area is progressing in a promising direction. Tam et al [71] proposed an evaluation framework aimed at enhancing reliability, generalizability, and applicability in human evaluation practices. Similarly, Van der Lee et al [67] introduced a set of best practices for the manual review of the automatically generated text, contributing to the development of more structured and consistent evaluation methodologies.

#### Challenges in Evaluating the Robustness of Model Performance

Assessing model robustness is crucial, as clinical settings differ in patient populations, writing styles, and medical practices. However, several issues were identified in the studies reviewed, particularly regarding the depth and comprehensiveness of the evaluation processes.

The sample sizes used in human evaluation were limited. Most studies assessed model performance on as few as 2 to 50 documents. Since LLMs generate nondeterministic outputs, they can vary between each iteration [72]. A previous work by Tam et al [71] suggests that at least 130 documents should be evaluated when testing clinical decision support tools to enable meaningful performance assessments.

In addition, model generalizability, referring to the model’s ability to perform effectively across diverse clinical settings, populations, or conditions beyond those on which it was originally trained [55], was frequently overlooked in the analyzed studies. External validation, essential for detecting overfitting and assessing cross-site transportability [73], was only reported by Kondadadi et al [35] and Dai et al [46]. Similarly, subgroup performance analysis was conducted in another 2 studies: Liang et al [29] evaluated cancer stage-specific performance using subpopulation testing, and Van Veen et al [47] conducted out-of-distribution testing, which involves evaluating the model data on underrepresented samples of the dataset [51]. Additionally, 5-fold cross-validation, which involves partitioning the training data into different subsets to ensure consistent model performance, was only used by Li et al [21].

Assessing model generalizability involves understanding how data variability affects model performance [74]. Distribution shifts in training data can lead to underperformance in underrepresented populations while causing overfitting in overrepresented subgroups [51]. As such, while models may demonstrate high performance within their training environments, their ability to generalize to broader clinical settings remains uncertain.

To ensure robustness, future research should prioritize generalizability assessments, including rigorous out-of-distribution testing and subcategory analysis within the same dataset, even when access to multiple external datasets is limited. Moreover, systematic reporting of demographic characteristics is essential for enhancing transparency and ensuring models are evaluated across diverse populations (Textbox 2) [54,66,75,76].

#### Limitations in Addressing the Clinical Impact of Summarization Models

##### Clinical Utility

Few studies assessed the clinical impact of LLM-based summarization. Wu et al [28] evaluated perceived benefits by clinicians, focusing on estimated time savings. Cai et al [44] examined the ease of revision, while Zhao et al [43] assessed the model’s clinical utility.

LLMs are expected to reduce clinician workload and improve information synthesis, raising high adoption expectations [75]. However, beyond assessing their technical performance, a thorough evaluation of both their utility and risks is crucial to inform responsible implementation and compliance with legal standards [77,78]. Future studies should determine whether summarization systems effectively serve their intended clinical purpose [77]. Additionally, since all studies included in this review were retrospective, prospective studies could provide a deeper understanding of the actual impact of LLM-based summarization models in clinical workflows [56].

##### Patient Safety Risks

The safety and potential clinical harm of evaluated models remained frequently unaddressed. Failure analysis was conducted in only 20% (n=6) of studies, with Van Veen et al [1] being the only study to examine patient safety risks.

Without a clear understanding of failure patterns, it becomes inherently difficult to assess their impact on clinical decision-making and patient safety, as does the development of effective safeguards to mitigate potential risks. While recent studies have attempted to establish taxonomies for hallucinations [79,80], failures may be inherently task-specific, underscoring the need for systematic error analysis and categorization based on existing research. Additionally, their impact on patient safety should be evaluated using appropriate risk analysis methods (Textbox 2).

Although no standardized risk assessment framework currently exists, a step toward addressing this gap is the risk matrix-based evaluation framework introduced by Asgari et al [81], which provides a structured methodology for categorizing and quantifying errors, enabling a systematic assessment of their impact on patient safety. Establishing standardized methodologies for failure analysis and risk assessment will be essential to ensure the safe and effective deployment of these models in clinical practice.

##### Bias and Fairness

LLMs risk exacerbating health disparities [82] as they absorb intrinsic biases during training on diverse data sources. These biases can manifest as harms in specific downstream tasks, impacting clinical decision-making [5,10,54,60,76,83]. For example, Zack et al [84] identified stereotypical demographic representations in LLM-generated diagnostic and treatment recommendations related to sex, ethnicity, and race. As a generative process, abstractive summarization also raises the potential to perpetuate these biases [83].

Despite these concerns, no study reported a biased assessment. Future research should use diverse datasets, prioritize bias detection, particularly in identifying discriminative language in model outputs, and develop mitigation strategies to ensure fairness in clinical decision support (Textbox 2) [54,75,76].

##### Data Privacy and Security

Ensuring data privacy and security is essential for ethical and legal compliance in research, and to enable valid and reproducible studies that can inform the responsible adoption of LLMs for clinical summarization. However, several studies used proprietary models to process patient datasets, raising concerns about data privacy and regulatory compliance.

For example, Van Veen et al [1], Ma et al [36], and Li et al [21] used the MIMIC series, however, only Van Veen et al [1] explicitly reported using the Azure OpenAI API. This raises concerns as the PhysioNet Credentialed Data Use Agreement explicitly prohibits sharing credentialed datasets (eg, MIMIC-III, MIMIC-IV, MIMIC-CXR) with third-party AI services, such as OpenAI APIs [85]. Chien et al [39] and Wang et al [41] used deidentified proprietary datasets. However, Wang et al [41] used these datasets without IRB approval, raising ethical and regulatory concerns regarding research oversight, patient privacy, and compliance with data protection standards.

Model selection must balance multiple factors, including performance, regulatory compliance, ethical considerations, and socioeconomic factors. Proprietary models, such as GPT-4, are attractive as they offer strong performance, ease of use, and cost-efficiency. However, there are currently there are currently no clear regulatory guidelines or ethical consensus on handling patient information in private models [5,86].

While Ma et al [36] argue that deidentification sufficiently protects patient privacy, studies have demonstrated that deidentified data could be reidentified, raising concerns about its reliability as a sole privacy safeguard [87]. Despite ongoing advancements in data protection methods, vulnerabilities persist, with emerging privacy attacks continuously exposing weaknesses in data protection measures [88].

Given these challenges, future research must explicitly disclose data-sharing practices, ensure compliance with legal agreements, and adopt privacy-preserving strategies, such as on-premises deployment (Textbox 2). Locally installed open-source solutions provide a controlled environment tailored to institutional needs, ensuring strict data privacy compliance while supporting valid, reproducible studies that can lead to practical solutions for reducing clinicians’ workload.

##### Technological and Cost Requirements

The successful deployment of LLMs also depends on their computational resource requirements and cost implications. While most studies reported hardware and memory requirements, none provided a detailed cost analysis.

Without these insights, health care institutions may struggle to assess the feasibility of implementing LLM-based solutions. Future research should go beyond hardware specifications to evaluate the economic impact of deployment to ensure that LLM adoption is both technically and financially sustainable in clinical settings (Textbox 3).

### Strengths and Limitations

The data extraction process was conducted independently by 3 authors, which enhances the accuracy and reliability of the results. By aligning with the PRISMA-ScR checklist, we ensure transparency throughout the review, allowing for a coherent and well-documented process.

However, several limitations must be acknowledged. First, to finalize the data extraction table, we randomly assessed 3 studies, following the methodology outlined by Pollock et al [89]. This may have introduced bias in defining extracted variables. Second, certain aspects of data extraction were not fully explored in this study, such as the architecture types of models and their performance. This decision was made to maintain a broader focus rather than a highly technical approach. Additionally, providing a detailed performance evaluation was not meaningful due to the limitations mentioned in the discussion. Third, as authors were not contacted, reliance on published information alone may have introduced bias, particularly where methodological details were incomplete or underreported. Finally, the rapid pace of new research may result in some emerging studies being missed, and relevant sources may be inaccessible due to publication bias or their status as preprints.

### Conclusions

This scoping review highlights key barriers to translating research advancements into practical applications, indicating that the field is still in its early stages. Research remains limited in scope, often shaped by dataset availability rather than explicitly guided by clinical needs, leaving many potential applications unexplored. Performance assessments frequently lack reliability and robustness, making it difficult to accurately evaluate model effectiveness. Furthermore, clinical impact evaluations remain insufficient, raising concerns about model utility, potential risks, fairness, data privacy, and broader technological and cost implications.

To advance this field, future research must broaden its scope, strengthen methodological transparency, and improve the reliability of evaluation frameworks. Additionally, enhancing model robustness and conducting comprehensive clinical impact assessments will be essential for determining the practical value of LLM-based summarization.

## Appendix

Multimedia Appendix 1 PRISMA-ScR (Preferred Reporting Items for Systematic Reviews and Meta-Analyses extension for Scoping Reviews) checklist.

Multimedia Appendix 2 Search queries from databases (as of June 18, 2024).

Multimedia Appendix 3 Data extraction table.

Multimedia Appendix 4 Summary of input sources and corresponding summarization objectives for each publication.

Multimedia Appendix 5 Additional information on results: test set size and specific evaluation metrics reported for each publications.

## Abbreviations

API: application programming interface
EHR: electronic health record
IRB: institutional review board
LLM: large language model
MIMIC: Medical Information Mart for Intensive Care
PRISMA: Preferred Reporting Items for Systematic Reviews and Meta-Analyses
PRISMA-ScR: Preferred Reporting Items for Systematic Reviews and Meta-Analyses extension for Scoping Reviews
TRIPOD: Transparent Reporting of a multivariable prediction model for Individual Prognosis or Diagnosis
