# Supplementary material for: Scientific Evidence for Clinical Text Summarization Using Large Language Models: Scoping Review
Source: J Med Internet Res. 2025 May 15;27:e68998. doi: 10.2196/68998 (PMC12123242; doi:10.2196/68998)
Supplement: Multimedia Appendix 2 [file jmir_v27i1e68998_app2.docx]

**Multimedia Appendix 2 : Search strategy**

| **Database** | **Exact Query Search** |
| --- | --- |
| Embase (Excerpta Medica Database) | (summarization: ti, ab, kw OR summarisation: ti, ab, kw) AND ('generative artificial intelligence': ti, ab, kw OR 'generative ai': ti, ab, kw OR 'generative model*': ti, ab, kw OR 'language model*': ti, ab, kw OR llm*: ti, ab, kw OR 'autoregressive model*': ti, ab, kw OR 'transformer*': ti, ab, kw OR 'generative pre-trained transformer*': ti, ab, kw OR gpt*: ti, ab, kw OR chatgpt*: ti, ab, kw OR llama*: ti, ab, kw OR mistral*: ti, ab, kw) AND (medical: ti, ab, kw OR clinical: ti, ab, kw OR medicine: ti, ab, kw OR health: ti, ab, kw OR healthcare: ti, ab, kw OR hospital: ti, ab, kw OR patient*: ti, ab, kw) |
| PubMed (U.S. National Library of Medicine) | ("Summarization"[Title/Abstract] OR "Summarisation"[Title/Abstract]) AND ("Generative Artificial Intelligence"[Title/Abstract] OR "Generative AI"[Title/Abstract] OR "generative model*"[Title/Abstract] OR "language model*"[Title/Abstract] OR "llm"[Title/Abstract] OR "autoregressive model*"[Title/Abstract] OR "transformer*"[Title/Abstract] OR "generative pre-trained transformer*"[Title/Abstract] OR "gpt"[Title/Abstract] OR "chatgpt*"[Title/Abstract] OR "llama*"[Title/Abstract] OR "mistral*"[Title/Abstract]) AND ("medical"[Title/Abstract] OR "clinical"[Title/Abstract] OR "medicine"[Title/Abstract] OR "health"[Title/Abstract] OR "healthcare"[Title/Abstract] OR "hospital"[Title/Abstract] OR "patient*"[Title/Abstract]) AND 2019/01/01:3000/12/31[Date - Publication] |
| Web of Science Core Collection | (Summarization OR Summarisation) AND ("Generative Artificial Intelligence" OR "Generative AI" OR "Generative model*" OR "Language model*" OR LLM* OR "Autoregressive Model*" OR "Transformer*" OR "Generative Pre-trained Transformer*" OR GPT* OR ChatGPT* OR Llama* OR Mistral*) AND (medical OR clinical OR medicine OR health OR healthcare OR hospital OR patient*) (Abstract) OR (Title) |
| IEEE Xplore Digital Library | (("Summarization" OR "Summarisation") AND ("Generative Artificial Intelligence" OR "Generative AI" OR "Generative model" OR "Language model" OR "LLM" OR "Autoregressive Model" OR "Transformer" OR "Generative Pre-trained Transformer" OR "GPT*" OR "ChatGPT" OR "Llama*" OR "Mistral*") AND ("medical" OR "clinical" OR "medicine" OR "health" OR "healthcare" OR "hospital" OR "patient")) |
| ACM Digital Library | [[Abstract: summarization] OR [Abstract: summarisation]] AND [[Abstract: "generative artificial intelligence"] OR [Abstract: "generative ai"] OR [Abstract: "generative model*"] OR [Abstract: "language model*"] OR [Abstract: llm*] OR [Abstract: "autoregressive model*"] OR [Abstract: "transformer*"] OR [Abstract: "generative pre-trained transformer*"] OR [Abstract: gpt*] OR [Abstract: chatgpt*] OR [Abstract: llama*] OR [Abstract: mistral*]] AND [[Abstract: medical] OR [Abstract: clinical] OR [Abstract: medicine] OR [Abstract: health] OR [Abstract: healthcare] OR [Abstract: hospital] OR [Abstract: patient*]] AND [E-Publication Date: (01/01/2019 TO *)] |

**Table 2.** Search Queries from Databases (as of June 18, 2024)
