# Supplementary material for: Scientific Evidence for Clinical Text Summarization Using Large Language Models: Scoping Review
Source: J Med Internet Res. 2025 May 15;27:e68998. doi: 10.2196/68998 (PMC12123242; doi:10.2196/68998)
Supplement: Multimedia Appendix 4 [file jmir_v27i1e68998_app4.docx]

**Multimedia Appendix 4 : Summary of input sources and corresponding summarization objectives for each publication.**

| Text corpus type | | | Summarization objective | References |
| --- | --- | --- | --- | --- |
|  | **Radiology reports (57%, 17/30)** | | | |
|  |  | Findings section of radiology report | To generate the impression section | [1–10] |
|  |  | Findings section of radiology report (+/- background section) | To generate the impression section | [11,12] |
|  |  | Findings section of radiology report | To generate "*a few significant impressions*" | [13] |
|  |  | Findings section of echography report | To generate "*a few critical diagnosis results*" | [13] |
|  |  | Background section + findings sections of Structured Oncology Reporting (SOR) | To generate the conclusion section of SOR | [14] |
|  |  | Diagnosis finding section of radiology report including : (1) the examination method used by the radiologist; (2) the body parts of the patient examined by the radiologist; (3) a description of the findings of the examined disease; and (4) a focused description of the abnormalities. | To generate the diagnostic opinions section | [15] |
|  |  | Neuroradiology reports | To generate single and longitudinal aneurysm imaging reports | [16] |
|  |  | Unstructured finding section of radiology report | To structure the unstructured findings section | [17] |
|  | **Progress notes (7%, 2/30)** | | |  |
|  |  | (1)Assessment section only (ASSMT), (2) Assessment and Subjective sections | To generate the plan section (list of problem) | [18] |
|  |  | Progress notes | To generate the corresponding problem list | [6] |
|  | **Patient form (3%, 1/30)** | | |  |
|  |  | Patient symptoms intake forms | To generate a summary of patient symptom form | [19] |
|  | **Various (23%, 7/30)** | | |  |
|  |  | Nursing notes, nursing other notes, physician notes, radiology notes, respiratory notes, case management notes, consult notes, discharge summaries, ECG notes, echo notes, general notes, nutrition notes, pharmacy notes, rehab services notes, and social work notes | To summarize clinical notes without noise and unnecessary information | [20] |
|  |  | Nursing notes, Demographic tables, Weight tables | Task 1: To generate a structured summary of text notes to inform nutrition status of residents | [21] |
|  |  |  | Task2 : To generate a list of malnutrition risk factors |  |
|  |  | Descriptive (Admission diagnosis, admission and discharge times, admission and discharge location, record of death, event times, insurance, religion, age, gender, marital status, and ethnicity) Interventions (Procedures performed such as dialysis and imaging studies) Notes (Echo, ECG, nursing, physician, rehab, case management, respiratory, general, nutrition, consult, social work, pharmacy, radiology, other, and discharge summary) Reports (Electrocardiogram and imaging studies) | To generate the hospital course section of the discharge summary | [22] |
|  |  | Descriptive (Age, sex, marital status, race, mortality status) Encounters (Admission date, admission diagnoses (ICD-10 code and description), discharge date, discharge disposition) Free text documents (Admission notes, emergency department provider notes, progress notes, consult notes, operative reports, pathology reports, radiology reports, discharge summaries) Measurements (Laboratory results (LOINC), vital signs) | To generate the hospital course section of the discharge summary | [23] |
|  |  | Free form notes of admission, history of medical treatment, nursing observation, pharmacy | To generate the critical diagnoses of the patient | [13] |
|  |  | In patient record (Admission records, war round records, course/surgery record) | To generate the complete discharge summaries | [24] |
|  |  | Discharge summaries, radiology, hematology, biopsy, urology and immunology report | To summarize key points of the report | [25] |
|  | **Unclear / Not mentioned (17%, 5/30)** | | |  |
|  |  | *« Notes associated with hospital discharge summaries »* | To generate a patient-friendly summary of discharge summary | [26] |
|  |  | *« EHR notes »* | To generate the patient after-visit summary | [27] |
|  |  | Not explicitly stated. Examples are given : nursing progress note, admission summary, radiology reports | To generate the brief hospital course section of discharge summaries | [28] |
|  |  | Not mentioned | Discharge diagnosis | [29] |
|  |  | Clinical scenarios in note format​ | To generate clinical letters & management plans | [30] |

1. Cai X, Liu S, Han J, Yang L, Liu Z, Liu T. ChestXRayBERT: A Pretrained Language Model for Chest Radiology Report Summarization. *IEEE Trans Multimed*. 2023;25:845-855. doi:10.1109/TMM.2021.3132724

2. Chuang YN, Tang R, Jiang X, Hu X. SPeC: A Soft Prompt-Based Calibration on Performance Variability of Large Language Model in Clinical Notes Summarization. *J Biomed Inform*. 2024;151:104606. doi:10.1016/j.jbi.2024.104606

3. Helwan A, Azar D, Ozsahin DU. Medical Reports Summarization Using Text-To-Text Transformer. In: *2023 Advances in Science and Engineering Technology International Conferences (ASET)*. ; 2023:01-04. doi:10.1109/ASET56582.2023.10180671

4. López-Úbeda P, Martín-Noguerol T, Díaz-Angulo C, Luna A. Evaluation of large language models performance against humans for summarizing MRI knee radiology reports: A feasibility study. *Int J Med Inf*. 2024;187:105443. doi:10.1016/j.ijmedinf.2024.105443

5. Ma C, Wu Z, Wang J, et al. An Iterative Optimizing Framework for Radiology Report Summarization with ChatGPT. *IEEE Trans Artif Intell*. Published online 2024:1-12. doi:10.1109/TAI.2024.3364586

6. Van Veen D, Van Uden C, Blankemeier L, et al. Adapted large language models can outperform medical experts in clinical text summarization. *Nat Med*. 2024;30(4):1134-1142. doi:10.1038/s41591-024-02855-5

7. Van Veen D, Van Uden C, Attias M, et al. RadAdapt: Radiology Report Summarization via Lightweight Domain Adaptation of Large Language Models. In: Demner-fushman D, Ananiadou S, Cohen K, eds. *The 22nd Workshop on Biomedical Natural Language Processing and BioNLP Shared Tasks*. Association for Computational Linguistics; 2023:449-460. doi:10.18653/v1/2023.bionlp-1.42

8. Alambo A, Banerjee T, Thirunarayan K, Cajita M. Improving the Factual Accuracy of Abstractive Clinical Text Summarization using Multi-Objective Optimization. *Annu Int Conf IEEE Eng Med Biol Soc IEEE Eng Med Biol Soc Annu Int Conf*. 2022;2022:1615-1618. doi:10.1109/EMBC48229.2022.9871798

9. Jiang Z, Cai X, Yang L, et al. Learning to Summarize Chinese Radiology Findings With a Pre-Trained Encoder. *IEEE Trans Biomed Eng*. 2023;70(12):3277-3287. doi:10.1109/TBME.2023.3280987

10. Yan A, McAuley J, Lu X, et al. RadBERT: Adapting Transformer-based Language Models to Radiology. *Radiol Artif Intell*. 2022;4(4):e210258. doi:10.1148/ryai.210258

11. Kondadadi R, Manchanda S, Ngo J, McCormack R. Optum at MEDIQA 2021: Abstractive Summarization of Radiology Reports using simple BART Finetuning. In: Demner-Fushman D, Cohen KB, Ananiadou S, Tsujii J, eds. *Proceedings of the 20th Workshop on Biomedical Language Processing*. Association for Computational Linguistics; 2021:280-284. doi:10.18653/v1/2021.bionlp-1.32

12. Dai S, Wang Q, Lyu Y, Zhu Y. BDKG at MEDIQA 2021: System Report for the Radiology Report Summarization Task. In: Demner-Fushman D, Cohen KB, Ananiadou S, Tsujii J, eds. *Proceedings of the 20th Workshop on Biomedical Language Processing*. Association for Computational Linguistics; 2021:103-111. doi:10.18653/v1/2021.bionlp-1.11

13. Zhu Y, Yang X, Wu Y, Zhang W. Leveraging Summary Guidance on Medical Report Summarization. *IEEE J Biomed Health Inform*. 2023;27(10):5066-5075. doi:10.1109/JBHI.2023.3304376

14. Liang S, Kades K, Fink M, et al. Fine-tuning BERT Models for Summarizing German Radiology Findings. In: Naumann T, Bethard S, Roberts K, Rumshisky A, eds. *Proceedings of the 4th Clinical Natural Language Processing Workshop*. Association for Computational Linguistics; 2022:30-40. doi:10.18653/v1/2022.clinicalnlp-1.4

15. Zhao S, Li Q, Yang Y, Wen J, Luo W. From Softmax to Nucleusmax: A Novel Sparse Language Model for Chinese Radiology Report Summarization. *ACM Trans Asian Low-Resour Lang Inf Process*. 2023;22(6). doi:10.1145/3596219

16. Chien A, Tang H, Jagessar B, et al. AI-Assisted Summarization of Radiologic Reports: Evaluating GPT3davinci, BARTcnn, LongT5booksum, LEDbooksum, LEDlegal, and LEDclinical. *AJNR Am J Neuroradiol*. 2024;45(2):244-248. doi:10.3174/ajnr.A8102

17. Ajad A, Saini T, Niranjan KM. Rad-Former: Structuring Radiology Reports using Transformers*. In: *2023 5th International Conference on Recent Advances in Information Technology (RAIT)*. ; 2023:1-6. doi:10.1109/RAIT57693.2023.10127096

18. Gao Y, Miller T, Xu D, Dligach D, Churpek MM, Afshar M. Summarizing Patients’ Problems from Hospital Progress Notes Using Pre-trained Sequence-to-Sequence Models. *Proc COLING Int Conf Comput Linguist*. 2022;2022:2979-2991. Accessed January 1, 10AD. https://pubmed.ncbi.nlm.nih.gov/36268128/

19. Wu DJ, Bibault JE. Pilot applications of GPT-4 in radiation oncology: Summarizing patient symptom intake and targeted chatbot applications. *Radiother Oncol J Eur Soc Ther Radiol Oncol*. 2024;190:109978. doi:10.1016/j.radonc.2023.109978

20. Li Q, Ma H, Song D, Bai Y, Zhao L, Xie K. Early prediction of sepsis using chatGPT-generated summaries and structured data. *Multimed TOOLS Appl*. Published online 2024. doi:10.1007/s11042-024-18378-7

21. Alkhalaf M, Yu P, Yin M, Deng C. Applying generative AI with retrieval augmented generation to summarize and extract key clinical information from electronic health records. *J Biomed Inform*. Published online 2024:104662. doi:10.1016/j.jbi.2024.104662

22. Hartman V, Campion TR. A Day-to-Day Approach for Automating the Hospital Course Section of the Discharge Summary. *AMIA Jt Summits Transl Sci Proc AMIA Jt Summits Transl Sci*. 2022;2022:216-225. https://pubmed.ncbi.nlm.nih.gov/35854728/

23. Hartman VC, Bapat SS, Weiner MG, Navi BB, Sholle ET, Campion TR Jr. A method to automate the discharge summary hospital course for neurology patients. *J Am Med Inform Assoc JAMIA*. 2023;30(12):1995-2003. doi:10.1093/jamia/ocad177

24. Wang H, Wu W, Dou Z, He L, Yang L. Performance and exploration of ChatGPT in medical examination, records and education in Chinese: Pave the way for medical AI. *Int J Med Inf*. 2023;177:105173. doi:10.1016/j.ijmedinf.2023.105173

25. Vinod P, Safar S, Mathew D, Venugopal P, Joly LM, George J. Fine-tuning the BERTSUMEXT model for Clinical Report Summarization. In: *2020 International Conference for Emerging Technology (INCET)*. ; 2020:1-7. doi:10.1109/INCET49848.2020.9154087

26. Goswami J, Prajapati K, Saha A, Saha A. Parameter-efficient fine-tuning large language model approach for hospital discharge paper summarization. *Appl SOFT Comput*. 2024;157. doi:10.1016/j.asoc.2024.111531

27. Cai P, Liu F, Bajracharya A, et al. Generation of Patient After-Visit Summaries to Support Physicians. In: Calzolari N, Huang CR, Kim H, et al., eds. *Proceedings of the 29th International Conference on Computational Linguistics*. International Committee on Computational Linguistics; 2022:6234-6247. Accessed July 24, 2024. https://aclanthology.org/2022.coling-1.544

28. Searle T, Ibrahim Z, Teo J, Dobson RJB. Discharge summary hospital course summarisation of in patient Electronic Health Record text with clinical concept guided deep pre-trained Transformer models. *J Biomed Inform*. 2023;141:104358. doi:10.1016/j.jbi.2023.104358

29. Chen YP, Chen YY, Lin JJ, Huang CH, Lai F. Modified Bidirectional Encoder Representations From Transformers Extractive Summarization Model for Hospital Information Systems Based on Character-Level Tokens (AlphaBERT): Development and Performance Evaluation. *JMIR Med Inform*. 2020;8(4):e17787. doi:10.2196/17787

30. Caterson J, Ambler O, Cereceda-Monteoliva N, Horner M, Jones A, Poacher AT. Application of generative language models to orthopaedic practice. *BMJ Open*. 2024;14(3):e076484. doi:10.1136/bmjopen-2023-076484
