# Supplementary material for: Scientific Evidence for Clinical Text Summarization Using Large Language Models: Scoping Review
Source: J Med Internet Res. 2025 May 15;27:e68998. doi: 10.2196/68998 (PMC12123242; doi:10.2196/68998)
Supplement: Multimedia Appendix 5 [file jmir_v27i1e68998_app5.docx]

**Multimedia Appendix 5 : Supplementary information on results : test set size and specific evaluation metrics reported for each publication.**

– : denotes unreported information

**Metrics Abbreviations:**

ROUGE Recall-Oriented Understudy for Gisting Evaluation

BLEU Bilingual Evaluation Understudy

SARI Sentence-level Accuracy and Recall Index

WC Word Count

WER Word Error Rate

SER Sentence Error Rate

Sent. Θ  Sentence Embedding Similarity

CUI Concept Unique Identifier

METEOR Metric for Evaluation of Translation with Explicit ORdering,

ROC Receiver Operating Characteristic

TRR Test Reduction Ratio

ELFA Entity level factual accuracy

**Table 4.** Overview of studies incorporating quantitative analysis (column 2-3) and human evaluation (column 4-5).

| Authors | Test Set Size | Metrics | Test Set Size | Metrics |
| --- | --- | --- | --- | --- |
| Cai et al. (2023) | 1829 | ROUGE-1/2/L, BLEU-1/2/3 | 30 | Readability, Factual correctness, Informativeness, Redundancy |
| Cai et al. (2022) | 1854 | Dale Chall readability scores, ROUGE-1/2/3/4/L, BERTScore, SARI | 18 | Adequacy, Faithfulness, Readability, Ease of Revision |
| Caterson et al. (2024) | 15 | Readable online tool | 15 | Accuracy, General tone, Insertion, Omission |
| Chen et al. (2020) | 589 | TRR, ROUGE-1/2/L, ROC curve | 246 | Sense |
| Chien et al. (2024) | 237 | TRR, ROUGE-1/2/L, BERTScore | 237 | Readability, Accuracy, Comprehensiveness, Redundancy |
| Gao et al. (2022) | 92 | ROUGE-L, BERTScore, Sent. θ, CUI F-score | 2 | -- |
| Goswami et al. (2024) | -- | ROUGE-L, BLEU, Loss evaluation | 100 | Human preference, precision |
| Hartman et al. (2023) | 660, 7111 | WC, WER, SER, ROUGE-1/2/L | 25 | Quality, Readability, Completeness, Factuality |
| Jiang et al. (2023) | 38000 | ROUGE-1/2/L, BLEU-4 | 60 | Readability, Redundancy, Informativeness, Factual correctness |
| Liang et al. (2022) | 1052 | ROUGE-1/L | 20 | Comprehensibility, Oncology correctness, Non-oncology correctness |
| López-Úbeda et al. (2024) | 2327 | ROUGE-L, BLEU-1/2/3/4, BARTScore, METEOR | 100 | Fluency, Coherence, Consistency, Relevance |
| Searle et al. (2023) | 4917 | ROUGE-2/Lsum, Extracted concept analysis | 40 | Coherance, Fluency, Consistency, Relevance |
| Van Veen et al. (2023) | -- | ROUGE-L, BLEU, BERTScore, RadGraph | -- | Correctness, Coherence, Ability to capture critical information |
| Van Veen et al. (2024) | 250 | ROUGE-L, BLEU, BERTScore, MEDCON | 100 | Completeness, correctness, conciseness |
| Vinod et al. (2020) | -- | ROUGE-1/2/L | -- | Effectiveness |
| Zhao et al. (2023) | 1029 | ROUGE-1/2/L, BLEU-1/2/3/4 | 30 | Clinical usability |

**Table 5.** Overview of studies incorporating human evaluation only.

| Authors | Test Set Size | Metrics | Test Set Size | Metrics |
| --- | --- | --- | --- | --- |
| Alkhalaf et al. (2024) |  |  | Task 1: 25/52  Task 2: 100 | -- |
| Li et al. (2024) |  |  | 100 | Readability, Faithfulness |
| Wang et al. (2023) |  |  | 20 | Logically, Quality (Lack of key information, Insufficient diagnosis, False information) |
| Wu et al. (2024) |  |  | 30 | Accuracy, Likelihood of adoption, Estimated clinical time saved |

**Table 6.** Overview of studies incorporating quantitative analysis only.

| **Authors** | **Test Set Size** | **Metrics** | Test Set Size | Metrics |
| --- | --- | --- | --- | --- |
| Ajad et al. (2023) | 1000 | BLEU-2/3/4/A |  |  |
| Alambo et al. (2022) | 6684 | ROUGE-1/2/L, ELFA |  |  |
| Chuang et al. (2024) | 200 | ROUGE-1/2/L, BERTScore |  |  |
| Dai et al. (2021) | 600 | ROUGE-1/L |  |  |
| Hartman et al. (2022) | 588 | ROUGE-1/2/L |  |  |
| Helwan et al. (2023) | 1140 | ROUGE-1/2/L/Lsum |  |  |
| Kondadadi et al. (2021) | 4000 | ROUGE-1/2/L |  |  |
| Ma et al. (2024) | 2182 | ROUGE-1/2/L, BERTScore, Factual Consistency |  |  |
| Yan et al. (2022) | 1000 | Text Reduction Ratio, ROUGE-1/2/L/L |  |  |
| Zhu et al. (2023) | 44524 | SummaC, QuestEval, ROUGE-1/2/L, BERTScore |  |  |
